# Picky eaters are rare: DNA-based blood meal analysis of *Culicoides* (Diptera: Ceratopogonidae) species from the United States

**DOI:** 10.1186/s13071-017-2099-3

**Published:** 2017-04-04

**Authors:** Matthew W. Hopken, Bonnie M. Ryan, Kathryn P. Huyvaert, Antoinette J. Piaggio

**Affiliations:** 1 USDA-APHIS-National Wildlife Research Center, 4101 Laporte Ave., Fort Collins, CO 80521 USA; 2 Department of Fish, Wildlife, and Conservation Biology, 1474 Campus Delivery Colorado State University, Fort Collins, CO 80523 USA; 3 Lake County Vector Control District, 410 Esplanade St, Lakeport, CA 95453 USA; 4 Present address: Department of Microbiology, Immunology, and Pathology, Colorado State University, 0922 Campus Delivery, Fort Collins, CO 80523-0922 USA

**Keywords:** Blood meal, Bluetongue virus, *Culicoides*, Epizootic hemorrhagic disease virus, Haemosporidia, Mitochondrial DNA, Vector

## Abstract

**Background:**

Biting midges in the genus *Culicoides* (Diptera; Ceratopogonidae) have been implicated in the transmission of a number of parasites and highly pathogenic viruses. In North America, the complete transmission cycles of many of these pathogens need further elucidation. One way to increase our knowledge about the evolution and ecology of *Culicoides* species and the pathogens they transmit is to document the diversity of vertebrate hosts that *Culicoides* feed upon. Our objective was to identify the diversity of *Culicoides* hosts in the United States.

**Results:**

We sequenced two vertebrate mitochondrial genes (cytochrome *c* oxidase subunit 1 and cytochrome *b*) from blood-engorged *Culicoides* to identify *Culicoides* species and their blood meals. We detected the mitochondrial DNA of 12 host species from seven different *Culicoides* species from three states. The majority of the identified blood meals were from the *C. variipennis* species complex in California. The hosts included both mammals and birds. We documented new host records for some of the *Culicoides* species collected. The majority of the mammalian hosts were large ungulate species but we also detected a lagomorph and a carnivore. The bird species that were detected included house finch and emu; the latter is evidence that the species in the *C. variipennis* species complex are not strictly mammalophilic.

**Conclusions:**

These results demonstrate that *Culicoides* will feed on multiple classes of vertebrates and may be more opportunistic in regards to host choice than previously thought. This knowledge can help with identification of susceptible host species, pathogen reservoirs, and new vector species which, in turn, will improve disease outbreak risk assessments.

**Electronic supplementary material:**

The online version of this article (doi:10.1186/s13071-017-2099-3) contains supplementary material, which is available to authorized users.

## Background

Understanding the sylvatic cycles and predicting epizootics of vector-borne pathogens requires knowledge about vector feeding behavior. Elucidating vector and host interactions helps with discovery of unknown vector species, susceptible hosts, reservoir species, and host-parasite-vector co-evolution, e.g. [1, 2]. One of the most critical ecological parameters for predicting vector-borne transmission is vector biting rates on susceptible hosts [3–5]. Thus, accurate host identification is a requirement for determining the impact these pathogens may have on human, wildlife, and livestock health.

Sylvatic transmission pathways of vector-borne pathogens are often difficult to delineate because they involve multiple host species. Many blood-feeding arthropods that serve as vectors, especially in the Diptera, have feeding behaviors that can be plastic in response to environmental change and host availability [6, 7]. For many vector species, we lack knowledge about the diversity of hosts they choose to bite [8–10]. Disentangling vector-borne pathogen transmission networks requires full characterization of the breadth of host species upon which vectors feed [11].

Biting midges of the genus *Culicoides* Latreille, 1809 (Diptera: Ceratopogonidae) are globally ubiquitous, except for New Zealand and Antarctica, and the group includes over 1400 described species [12, 13]. Females of most species are hematophagous and transmit a multitude of pathogenic viruses, filarial worms, and blood parasites to humans, livestock, and wildlife [14]. Among the most damaging *Culicoides*-borne pathogens are bluetongue virus (BTV), epizootic hemorrhagic disease virus (EHDV), vesicular stomatitis virus (VSV), African horse sickness (AHSV), and Schmallenberg virus (SBV) [13, 15, 16]. Each of these viruses causes outbreaks in wildlife and livestock globally that have been linked to severe economic losses due to both lost production and disease-related trade restrictions [17–20]. In North America, the most commonly encountered vector species of BTV is *C. sonorensis* Wirth & Jones, but the evidence for which species is the main vector of EHDV is ambiguous [21–24].

Multiple methods can be employed to identify the source of arthropod blood meals, but, to date, genetic techniques have proven to be the most accurate when applied to *Culicoides*, e.g. [25, 26]. In North America, three studies used molecular methods to identify the source of *Culicoides* blood meals [27–29]. However, these studies were conducted over limited geographic areas and the sample sizes were very small. Here, we expand our collective knowledge of *Culicoides* host choice by sequencing two mitochondrial DNA loci from blood-engorged *Culicoides* collected from three states, including one area where BTV is enzootic in both livestock and wildlife [30, 31]. The information gained from this study will help illuminate the diversity of *Culicoides* hosts, which will help us to better understand pathogen transmission across the livestock/wildlife interface, in addition to advancing our understanding of the natural history of this ubiquitous, abundant, and economically important group of insects.

## Methods

### Field collection and species identification

Blood-engorged *Culicoides* individuals were captured opportunistically as part of surveillance efforts, or for other studies, between 2011 and 2014 from three regions in the USA. Insects were captured with CDC mini light traps (Bioquip, USA) or New Jersey light traps baited with ultraviolet light and CO_2_. Insects were stored on dry ice immediately after capture then stored at -80 °C, or placed immediately in 99% ethanol. *Culicoides* were sorted from trap by-catch under stereomicroscopes on a cold plate (Bioquip, Rancho Domingo, California, USA). Species identification was first based on wing patterns following Wirth et al. [32]. For some subgenera and species groups, wing patterns are not species-diagnostic so we used combinations of morphological characters identified as diagnostic by Blanton & Wirth [33], Battle & Turner [34] and Holbrook et al. [35] to identify *Culicoides* species to the lowest possible taxon. We also included three blood-engorged individuals from the *C. sonorensis* USDA-ARS captivity colony to validate our blood meal identification assays. These individuals were fed domestic sheep (*Ovis aries* L.) blood.

### Molecular species identification

Morphological identification of the species within the *C. variipennis* species complex is not always straightforward as there are few, if any, discrete synapomorphies that define the species. In parts of each species’ range *C. occidentalis*, *C. sonorensis* and *C. variipennis* are sympatric. The females of the former two species are morphologically indistinguishable whereas the latter two species have differences in maxillary palp morphometrics which are not consistent as intraspecific variation leads to overlapping size ranges [35]. In an attempt to differentiate these sympatric sibling species, we sequenced 650 base pairs of the 5′ region of the mitochondrial cytochrome *c* oxidase subunit 1 gene (*cox*1). Currently no comprehensive database of the *cox*1 gene for North American *Culicoides* species exists so we developed our own *cox*1 sequence database that included 5 males of *C. occidentalis* from Lake County, California and two *C. sonorensis* females, one from the captive breeding colony maintained by the USDA-ARS in Manhattan, Kansas, and one collected along the front range of Northern Colorado, where *C. sonorensis* is allopatric. We also included three *C. variipennis* samples from three states, Indiana, Michigan and New Jersey, where *C. variipennis* is allopatric. These reference specimens were identified to species by slide mounting and following the key provided in Holbrook et al. [35].

Genomic DNA from individual blood-engorged females and their blood meals were extracted using the DNEasy blood and tissue kit (Qiagen, Hilden, Germany). Insects were extracted in a room dedicated to non-invasive and environmental DNA samples. Each specimen was placed in a sterile Petri dish and we separated the abdomen from the thorax with sterilized tools. The entire insect was placed in 300 μl of Qiagen buffer ATL. We then added 20 μl of proteinase K and incubated the samples overnight at 56 °C. The following day the supernatant was transferred to a new 1.7 ml tube, leaving the exoskeleton of the insect in the initial tube. We added absolute ethanol to the exoskeleton and stored it at -20 °C for slide-mounting for morphological species identification. We used an automated extraction protocol “Isolation of DNA from forensic casework samples part B (purification)” extraction protocol designed for the QIAmp DNA investigator kit (Qiagen, Germany) on the QIAcube (Qiagen, Germany) to extract DNA from the supernatant. The final elution volume was adjusted to 50 μl. An extraction blank containing only reagents was included with each extraction set to monitor for contamination. DNA extracts were stored at -20 °C until further processing.

We used primers from two different studies to sequence 650 bp of *cox*1, BFculicFm1 [36] and C1-N-2191 [37]. The gene was amplified using PCR in 25 μl reactions with 0.25 mM of each dNTP, 2 mM MgCl_2_, 0.4 uM of each primer, 2 units of Amplitaq Gold polymerase (Thermo Fisher, Waltam, Massachusetts, USA) and 1 μl of DNA extract. The PCR program we used had an initial denaturing step at 95 °C for 15 min then 40 cycles of 94 °C for 30 s, annealing at 54 °C for 30 s, extension at 72 °C for 1 min, and then a final extension of 72 °C for 5 min. Negative controls were included with each PCR to monitor contamination. PCR success was evaluated with 2% agarose gel stained with ethidium bromide. Successful reactions were cleaned up using ExoSAP-it (Affymetrix, Santa Clara, California, USA). Cycle sequencing was performed using BigDye v3.1 using the manufacturer’s recommended thermocycler program but with an annealing temperature of 52 °C. The cycle sequence products were purified with sephadex clean up using either PrepEase Sequencing Dye Clean-up Kit (Affymetrix) or 96-well filter plates (Whatman, Maidstone, UK). Sanger sequencing was performed on an Applied Biosystems Genetic Analyzer 3500xl (Life Technologies, USA).

Sequences were edited and aligned using CLUSTALW [38] implemented in GENEIOUS R7 (Biomatters, San Francisco, California, USA). We used a neighbor-joining (N-J) tree constructed in MEGA 6 [39, 40] to identify genetic clusters in the *C. variipennis* species complex that were associated with reference samples. One sequence from *C. reevesi* collected in California was included as an outgroup for the N-J tree. The tree was built using an uncorrected p-distance and branch support was evaluated using 1000 bootstrap replicates [41, 42].

### Blood meal identification

To identify the origins of *Culicoides* blood meals, we followed Pettersson et al. [26] and used the general primers COI_short_F/COI_short_R and L14841/H15149 to amplify around 350 bp of vertebrate cytochrome *c* oxidase subunit 1 (*cox*1) and cytochrome *b* (*cytb*), respectively [43, 44]. We first attempted to amplify *cox*1, but if this failed for samples we tried to amplify *cytb*. For *cox*1, the total reaction volume was 25 μl and contained 2.5 μl 10× buffer II, 1 mM of MgCl_2_, 0.1 mM of each dNTP, 0.4 mM of each primer, 2 units of Amplitaq Gold, and 2 μl of DNA extract. The thermocycler profile included an initial denaturing step at 95 °C for 15 min then 45 cycles of 94 °C for 30 s, annealing at 50 °C for 45 s, extension at 72 °C for 30 s and a final extension at 72 °C for 10 min. The *cytb* gene was amplified in a 25 μl reaction that contained 2.5 μl 10× buffer II, 1.5 mM of MgCl_2_, 0.15 mM of each dNTP, 0.4 mM of each primer, 2 units of Amplitaq Gold and 2 μl of DNA extract. The *cytb* thermocycler profile was identical to the one used for *cox*1 but had an annealing temperature of 52 °C. Negative controls were included with each PCR to monitor contamination. PCR success and sequencing methods were identical to those used for *Culicoides* species identification.

DNA sequences were edited and compared to GenBank *via* the BLAST function in GENEIOUS R7 (Biomatters, New Zealand), or to the Barcode of Life Database (BOLD; [45]) to determine species identification. We used a threshold of 98% to determine the species identification of vertebrate DNA sequences. If the sequences did not match to a species within this threshold on GenBank or BOLD then we identified it as the genus with the highest match. In some cases, known geographical distributions of hosts were used to assign the host to species (e.g. a sample identified as *Odocoileus* from California must be black-tailed deer (*Odocoileus hemionus* Rafinesque)). We tested for differences in blood meal sources between two groups identified as *C. occidentalis* and *C. variipennis* species complex using a *χ* ^2^ test.

## Results

We collected a total of 366 blood-engorged specimens from three states, New York, South Carolina, and California (Table 1; Additional file 1: Table S1). We were able to identify the sources of the blood meals from 199 blood engorged *Culicoides* from three states, which represented six morphologically identified species and one species complex (Table 1). The *C. variipennis* complex accounted for 183 of the 199 individuals (92%).

**Table 1:** Results from molecular blood meal analyses based on the cytochrome *c* oxidase I (*cox*1) and the cytochrome *b* (*cytb*) genes for *Culicoides* species in North America

			Mammals										Birds	
*Culicoides* species	State	*n*	Cattle	Dog	Goat	Donkey	Horse	Black-tailed Jackrabbit	Black-tailed deer	White-tailed deer	Sheep	Swine	Emu	House finch
*C. biguttatus*	New York	9								9				
*C. crepuscularis*	California	1												1
*C. reevesi*	California	1							1					
*C. stellifer*	New York	4								4				
*C. utahensis*	California	1							1					
*C. variipennis* species complex														
*C. occidentalis*	California	120	14	9	1	1	14	7	51		18	1	4	
*C. variipennis* complex^a^	California/ South Carolina	63	17	2		1	8	4	21		6	2	2	
Total		199	31	11	1	2	22	11	74	13	24	3	6	1

^a^ *Culicoides variipennis* complex includes individuals morphologically identified as belonging to the *C. variipennis* species complex, but precise species identification could not be made because no genetic data were available or they grouped in cluster B or C in the neighbor-joining tree (Fig. 1)

Presented in the table are *Culicoides* species, the state where collected, sample size (*n*), number of host species identifications from blood meals. The host species are: black-tailed deer (*Odocoileus hemionus*); black-tailed jackrabbit (*Lepus californicus*); cattle (*Bos taurus*); domestic dog (*Canis familiaris*); domestic goat (*Capra hircus*); domestic sheep (*Ovis aries*); donkey (*Equus asinus*); horse (*Equus. caballus*); swine (*Sus scofa*); white-tailed deer (*O. virginianus*); emu (*Dromaius novaehollandiae)*; house finch (*Haemorhous mexicanus*)

### *Culicoides* species identification

**Fig. 1:**
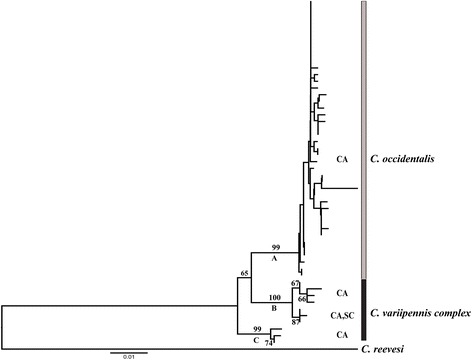
*Culicoides* spp. neighbor-joining tree constructed in MEGA6 using the uncorrected p-distance. The data for the tree were cytochrome *c* oxidase subunit 1 (*cox*1) sequences from *Culicoides variipennis* species complex collected in Northern California and South Carolina. Branch labels are statistical support generated by 1000 bootstrap replicates. Only values greater than 50% are shown. The labels on the right are species identification based on reference samples. The scale-bar represents the average number of base pair changes per site. Only two species could be identified, *C. occidentalis* (A) and all other *C. variipennis* species complex individuals (B and C). Cluster A contained the five male reference specimens identified as *C. occidentalis*. Cluster B contained the female reference specimens identified as both *C. sonorensis* and *C. variipennis*. Cluster C did not contain any reference specimens. We could not differentiate *C. sonorensis* and *C. variipennis* using *cox*1 thus we grouped clusters B and C under the *C. variipennis* complex. The abbreviations for the state from which the samples were collected are to the right of each cluster. *Abbreviations*: CA, California; SC, South Carolina

We successfully sequenced *cox*1 for 139 of the 183 blood-engorged *C. variipennis* complex individuals. There were 54 unique haplotypes (including reference samples) that fell into three well supported genetic clusters (Fig. 1). Cluster A is the largest (Fig. 1), with branch support of 99%, contained 43 haplotypes that accounted for 120 individuals and this group also included morphologically confirmed *C. occidentalis* reference samples. The other two well-supported clusters, B and C, (Fig. 1) contained 8 haplotypes and 3 haplotypes, respectively. The 16 individuals in cluster B were from California and South Carolina, and the three individuals in cluster C were from California. The *C. sonorensis* and *C. variipennis* reference samples grouped into cluster B while no reference samples grouped into cluster C. All the individuals in clusters B and C were morphologically identified as belonging to the *C. variipennis* complex.

### Blood meal identification

Our approach to blood meal identification was successful in that we identified the blood source of the three captive *C. sonorensi*s as domestic sheep, the known host species from which the blood was collected. We also successfully identified the sources of blood meals from199 wild-caught individuals (Table 1; Additional file 1: Table S1). Of the 199 successfully identified blood meals, 183 (92%) came from the *C. variipennis* species complex. Individuals identified as *C. occidentalis* using *cox*1 accounted for 120 of 183 *C. variipennis* complex blood meals. The other 63 blood meals were from *C. variipennis* complex individuals that were part of cluster B and C (*n* = 19), or for which we were unable to obtain molecular species ID (*n* = 44). From here on we refer to these individuals as the *C. variipennis* species complex. There were no statistical differences between the blood meal sources identified from *C. occidentalis* and *C. variipennis* species complex (*χ* ^2^ = 11.2, *df* = 8, *P* = 0.192). Most of the *C. occidentalis* and *C. variipennis* species complex blood meals were from large ungulates and these included both wild species, black-tailed deer (*n* = 72; 39%), 5 domestic mammals (*n* = 80; 44%), and swine (*Sus scrofa* L.; *n* = 3; 2%) which could be either feral or domestic (Table 1). Eleven of the 183 (6%) were from black-tailed jackrabbit (*Lepus californicus* Gray) and 11 (6%) from domestic dog (*Canis familiaris* L.). The remaining six individuals (3%) fed upon emu (*Dromaius novaehollandiae* Latham).

Sixteen of the 199 (8%) identified blood meals were from *Culicoides* species not in the *C. variipennis* species complex: *C. bigutattus* (Coquillett)*, C. crepuscularis* Malloch, *C. reevesi* Wirth, *C. stellifer* (Coquillett), and *C. utahensis* Fox. All of the non-*variipennis* blood meals were from deer (*Odocoileus* spp.), except one *C. crepuscularis* that fed on a house finch (*Haemorhous mexicanus* (Müller)). We also detected human DNA in some of the samples (not included in the sample size of 199). These samples were excluded from further analysis as determining whether it was a blood meal taken from a human or contamination was not possible. Despite using both genetic loci, we only had a 54% success rate which was due to improper storage conditions for one batch of insects that resulted in DNA degradation in the majority of samples.

## Discussion

The main finding from this study was that North American *Culicoides* host choice was broader than previously understood. Through the use of genetic approaches, we identified a diversity of known hosts, including some that are highly susceptible to infection with BTV and EHDV, and expanded the catalog of hosts through detection of previously undocumented vertebrate host species. Our results represent the most comprehensive dataset to date regarding Nearctic *Culicoides* host choice. The value of these data cannot be understated as one of the most important shortcomings in our knowledge of *Culicoides* biology is the lack of data regarding trophic behavior and host selection across diverse habitats [23, 46].

### *Culicoides* species identification

The results from molecular identification of the three species in the *C. variipennis* species complex suggested that identifying species based on sampling location and breeding habitat is not as straight-forward as previously suggested, e.g. [35]. Females collected for this study from California were initially identified as *C. sonorensis* or *C. occidentalis* based on sampling location (BMR, unpublished data). However, the molecular results identified almost all of the samples as *C. occidentalis* (*n* = 120). Thus, the approach suggested in Holbrook et al. [35] using location or breeding habitat did not work in this case but it does suggest that *C. sonorensis* is less abundant in Lake County, California than previously thought. Considering that this region has a high rate of BTV and EHDV transmission [31], and given the high occurrence of *C. occidentalis*, perhaps *C. occidentalis* should be investigated further as a potential vector. One other interesting point is that haplotypes from California and South Carolina clustered closely together and with the *C. variipennis* and *C. sonorensis* reference samples (Fig. 1, Cluster B). This lack of genetic differentiation between *C. sonorensis* and *C. variipennis* brings into question the utility of *cox*1 to discriminate between these two species, the diagnostic morphological traits, the range estimations, or the current taxonomy. To clarify the molecular taxonomy a multi-locus phylogenetic study is required to help refine the evolutionary relationships, distribution, and ecology of the *C. variipennis* species complex [24] as this study was not designed to answer such questions.

The high occurrence of *C. occidentalis* in our California sampling area was surprising as *C. sonorensis* is thought to be more broadly distributed and abundant [35, 47]. There are explanations for the discrepancy between our data and those collected previously. The first is that perhaps *cox*1 is not a good genetic locus for species identification*.* However, a more thorough sampling across the US has demonstrated that suggests *cox*1 can easily discriminate between *C. occidentalis* and the two other species in the complex [24]. Another explanation could be that *C. sonorensis* and *C. occidentalis* hybridize and the results herein are due to mitochondrial DNA introgression. Velten & Mullens [48] were able to successfully hybridize these two species in the laboratory and the cross of *C. sonorensis* male and *C. occidentalis* female had high reproductive success rates. Thus, if these two species hybridize in the wild then *C. occidentalis* mtDNA haplotypes could be present in *C. sonorensis* populations due to maternal inheritance. However, previous allozyme studies have not found natural hybrids in zones of sympatry in California [35, 47]. Despite some ambiguous species identifications, this study, along with a similar study of Palearctic *Culicoides* spp. Pettersson et al. [26], demonstrated the importance of combining molecular species identification of pathogen vectors to improve accuracy of associating blood meals with the correct species.

### Blood meal identification

Documenting the diversity of hosts in a particular area, including those hosts that have been catalogued previously, can help understand the influence of host availability and density on *Culicoides* host choice and pathogen transmission [5]. We identified the source of blood meals from 199 *Culicoides* that represented 12 different host species (Table 1, Fig. 1). Four of these mammalian species are susceptible to *Culicoides*-borne pathogens and have been previously identified as hosts for the *C. variipennis* species complex including: horse (*Equus caballus* L.), cattle (*Bos taurus* L.), domestic sheep and white-tailed deer (*O. virginianus* (Zimmerman)) [21, 49–51]. The other six mammalian species detected: domestic dog, domestic goat (*Capra hircus* L.), donkey (*E. asinus* L.), black-tailed jackrabbit, swine, and black-tailed deer, have not been documented as hosts of the *C. variipennis* complex until this study. The detection of blood meals from hosts that are susceptible to infection with BTV and EHDV is further evidence of the role that the *C. variipennis* species complex plays in pathogen transmission across the US and demonstrates the presence of potential reservoir species.

The results presented here, combined with previous findings, confirmed that the *C. variipennis* species complex will feed upon mammals other than large ungulates, indicating that they are opportunistic when choosing host species for blood meals. Tempelis & Nelson [29] used bait animals and immunological approaches to identify blood meals of the *C. variipennis* species complex in California, but could only distinguish hosts to taxonomic family (Bovidae and Leporidae). Our data substantiated their findings as we found 31 individuals had fed upon cattle (Bovidae), and we documented, for the first time, that this species complex feeds on black-tailed jackrabbits (Leporidae; Table 1). We were also able to document the first occurrence of the *C. variipennis* species complex feeding on domestic dog*.* Alexander et al. [52] reported that African carnivores can be infected with BTV. Our confirmation of *Culicoides* feeding upon a canid species and a wild leporid suggest that carnivores and other small mammals should be evaluated as potential contributors to the transmission cycles of BTV and EHDV.

The data we collected for this study provide the first evidence that the *C. variipennis* complex will take blood meals from black-tailed deer. Epidemiological data suggest that *C. variipennis* will bite black-tailed deer as Roug et al. [31] detected considerable numbers of black-tailed deer in northern California that were seropositive for both BTV and EHDV. Free movement of BTV between deer and livestock in the same area has been documented when large numbers of *C. variipennis* species complex individuals are present [30]. One remarkable point is that black-tailed deer are often asymptomatic when infected with BTV and EHDV such that detecting outbreaks in this host species can be challenging [53]. Asymptomatic hosts can facilitate movement of pathogens across the landscape, and may even facilitate overwintering of the viruses by maintaining viral infections through the months when vectors are not present. The combination of blood meal and epidemiological data provides additional evidence that black-tailed deer can contribute to maintenance of the BTV enzootic cycle in northern California.

The first evidence of the *C. variipennis* species complex feeding on a bird was discovered in this study (Table 1). Emus are not native to North America but there are commercial farms and hobbyists, including in northern California, that raise the species for meat or other purposes (California Department of Agriculture, personal communication). Our detection of *Culicoides* blood meals from emus was unexpected and these results counter the assumption that the species in the *C. variipennis* complex feed exclusively on mammals. In fact, considering the size of emus, one could hypothesize that host choice in the *C. variipennis* species complex is not necessarily driven by phylogeny but rather by factors associated with body size, such as the larger emissions of CO_2_ or volatile compounds. This hypothesis has been tested with mosquitoes and other *Culicoides* species and the strict mammal *versus* bird hypothesis was rejected in favor of body size-driven host choice [5, 54, 55]. One important point from these findings is that emus can be infected with avian blood parasites [56] and, when emus are imported, they should be screened for infection to limit the chances of introduction and potential transmission of foreign blood parasite species to North American poultry and wild birds through *Culicoides* bites [57].

We obtained blood meal identification for six other *Culicoides* species not in the *C. variipennis* complex (Table 1). *Culicoides biguttatus*, a common species east of the Mississippi River [33], had fed on white-tailed deer, which confirms previous findings [28, 58–60]. In fact, Smith et al. [60] documented *C. biguttatus* feeding on white-tailed deer in an area that is enzootic for both BTV and EHDV pointing to a potential role of this species as a vector. *Culicoides stellifer* is a common species across continental North America [33] that we also found had fed on white-tailed deer. This species has been documented as feeding on large ungulates and is suspected as playing a role in the transmission of EHDV [23, 58, 60, 61]. We collected one *C. crepuscularis,* a species that ranges across the continental US, and this individual had fed upon a house finch. This *Culicoides* species is known to prefer avian species and is also a vector for nematodes and avian blood parasites [62–64]. *Culicoides utahensis* is found only in the western US and is known to use deer as a blood meal source [65], which we confirmed. *Culicoides reevesi* is distributed in the southwestern US and, to date, has only been recorded feeding on humans [66]. We have added to the knowledge of this species by presenting the first documented case of it feeding upon black-tailed deer.

## Conclusion

Vector*-*borne pathogens are significant threats to the health and welfare of both humans and animals worldwide [67]. More investigations into the ecology of all *Culicoides* species, not just the currently known vectors, will lead to better predictions of disease outbreaks and their attendant negative impacts on animal and human populations. In the face of climate change, the distributions of vector species and pathogens are expected to change [68, 69]. The potential exists for *Culicoides-*borne pathogens restricted to warmer parts of the world to move north or south leading to devastating outbreaks [70–72]. With the continuous threat of the introduction of foreign pathogens, we must build a solid foundation of knowledge regarding *Culicoides* host choice so that we can develop better models to predict transmission and to develop methods to study and manage *Culicoides* and the pathogens they transmit. Our dataset is the most comprehensive blood meal analysis for North American *Culicoides* to date. One shortcoming of this study is that we used Sanger sequencing which needs high quality DNA and cannot identify mixed blood meals from multiple sources [4]. Applying next-generation sequencing technology could improve the success and sensitivity of *Culicoides* blood meal identification [73]. Nevertheless, we have demonstrated that molecular tools are important for gaining a solid knowledge base and developing methods to address some of the most pressing needs for managing *Culicoides*-borne diseases.

## Additional file

Additional file 1: Table S1. Summary of data for blood-engorged *Culicoides* species collected in North America. Provided are the collection locality information (State, county, latitude and longitude), year collected (year), *Culicoides* species, sample size at each locality (*n*), and identification of host using DNA-based blood meal analysis (Host ID). The host species are: black-tailed deer (*Odocoileus hemionus*); black-tailed jackrabbit (*Lepus californicus*); cattle (*Bos taurus*); domestic dog (*Canis familiaris*); domestic goat (*Capra hircus*); domestic sheep (*Ovis aries*); donkey (*Equus asinus*); horse (*Equus. caballus*); swine (*Sus scofa*); white-tailed deer (*O. virginianus*); emu (*Dromaius novaehollandiae*); house finch (*Haemorhous mexicanus*). We also included the cluster in the neighbor-joining tree that contained haplotypes from individuals at each location. If we could not obtain genetic information from individuals at that location this was also included. (XLSX 15 kb)

## Abbreviations

BTV: Bluetongue virus
EHDV: Epizootic hemorrhagic disease virus
